# The divergence and positive selection of the plant‐specific BURP‐containing protein family

**DOI:** 10.1002/ece3.1792

**Published:** 2015-11-02

**Authors:** Lihui Wang, Ningning Wu, Yan Zhu, Wanlu Song, Xin Zhao, Yaxuan Li, Yingkao Hu

**Affiliations:** ^1^ College of Life Sciences Capital Normal University Beijing 100048 China

**Keywords:** BURP proteins, functional divergence, hormone response, phylogenetic tree, positive selection, promoter region, segmental duplication, stress response, tandem duplication

## Abstract

BURP domain‐containing proteins belong to a plant‐specific protein family and have diverse roles in plant development and stress responses. However, our understanding about the genetic divergence patterns and evolutionary rates of these proteins remain inadequate. In this study, 15 plant genomes were explored to elucidate the genetic origins, divergence, and functions of these proteins. One hundred and twenty‐five BURP protein‐encoding genes were identified from four main plant lineages, including 13 higher plant species. The absence of BURP family genes in unicellular and multicellular algae suggests that this family (1) appeared when plants shifted from relatively stable aquatic environments to land, where conditions are more variable and stressful, and (2) is critical in the adaptation of plants to adverse environments. Promoter analysis revealed that several responsive elements to plant hormones and external environment stresses are concentrated in the promoter region of BURP protein‐encoding genes. This finding confirms that these genes influence plant stress responses. Several segmentally and tandem‐duplicated gene pairs were identified from eight plant species. Thus, in general, BURP domain‐containing genes have been subject to strong positive selection, even though these genes have conformed to different expansion models in different species. Our study also detected certain critical amino acid sites that may have contributed to functional divergence among groups or subgroups. Unexpectedly, all of the critical amino acid residues of functional divergence and positive selection were exclusively located in the C‐terminal region of the BURP domain. In conclusion, our results contribute novel insights into the genetic divergence patterns and evolutionary rates of BURP proteins.

## Introduction

The BURP domain‐containing gene family is only found in the plant kingdom. This family is defined by the conserved C‐terminal amino acid domain of BURP proteins and is named after the four members that were initially identified: BNM2, USP, RD22, and PG1*β* (Hattori et al. 1998). BNM2 is a microspore protein that is found in oilseed rape (*Brassica napus*) and was identified during microspore embryogenesis (Boutilier et al. 1994). USPs belong to a class of abundant, nonstorage seed proteins found in the field bean (*Vicia faba*) and are expressed during the early zygotic stages (Bassuner et al. 1998). RD22 is a dehydration‐responsive protein found in thale cress (*Arabidopsis thaliana*) (Yamaguchi‐Shinozaki and Shinozaki 1993b, 1994). PG1*β* is a noncatalytic *β*‐subunit of polygalacturonase isozyme I that is expressed during the ripening of tomatoes (*Solanum lycopersicum*) (Zheng & DellaPenna, 1992).

The phylogenetic analysis of the putative BURP domain‐containing proteins in soybean and other plant species resulted in BURP domain‐containing proteins being initially classified into four subfamilies: BNM2‐like, USP‐like, RD22‐like, and PG1*β*‐like (Granger et al. 2002). All of the identified members of each subfamily contain the BURP domain in the C‐terminal region. Within the BURP domain, several amino acid residues are substantially conserved; specifically, two cysteine (C) residues and four repeats of the CH (cysteine‐histidine) motif (CHX_10_CHX_23–27_CHX_23–26_CHX_8_W, where X is any random amino acid residue) (Ding et al. 2009). In addition, among BURP domain‐containing gene family members, the greatest variability occurs in regions that contain either a short conserved segment or a short segment together with optional repeat segments. Unlike other BURP proteins, BNM2‐like proteins lack repeat units. Both USP‐ and RD22‐like proteins are distinguished from other BURP proteins by a conserved region, which contains ~30 amino acid residues followed by a variable region. However, RD22‐like proteins contain repeat sequences in the variable region that are not found in similar variable regions of USP‐like proteins (Granger et al. 2002). PG1*β*‐like proteins differ to other BURP subfamilies because they contain multiple copies of a sequence segment with a 14‐amino acid sequence (e.g., FTNYGXXGNGGXXX, where X = any amino acid residue) in PG1 (Zheng & DellaPenna, 1992). In addition, several new members of the BURP domain‐containing gene family have been identified from various plant species, leading to the emergence of several new subfamilies (including BURP‐V, BURP‐VI, and BURP‐VII). This development has increased the complexity of the BURP phylogenetic tree (Ding et al. 2009; Xu et al. 2010; Gan et al. 2011; Shao et al. 2011; Matus et al. 2014).

Even though the BURP family is easily classified from sequence features, the function of most BURP domain‐containing genes has not been elucidated. Several members of this family make important contributions to plant development and metabolism. *PG1β* helps regulate pectin metabolism in ripening tomatoes by limiting the extent of pectin solubilization and depolymerization (Zheng et al. 1992; Watson et al. 1994). The overexpression of *OsBURP16*, which is the *β*‐subunit of polygalacturonase 1 in rice (*Oryza sativa*), is induced by salinity, cold, drought, and ABA (abscisic acid) treatment. The overexpression of *OsBURP16* in rice decreases pectin content and cell adhesion, while increasing abiotic stress sensitivity (Liu et al. 2014). SCB1 (seed coat BURP‐domain protein 1) in soybean (*Glycine max*) may contribute to seed coat formation by regulating the differentiation of seed coat parenchyma cells (Batchelor et al. 2002). OsRAFTIN1 is an anther‐specific protein in rice that is exclusively expressed in the tapetum during postmeiotic stages. OsRAFTIN1 helps transport sporopollenin from the tapetum to developing microspores via the Ubisch bodies. The suppressed expression of the OsRAFTIN1 may cause the nondehiscence and shortening of mature anthers, as well as pollen grain collapse (Wang et al. 2003). Another anther‐specific BURP protein, RA8, is specifically expressed in the tapetum, connective tissue, and endothecium, but not in pollen grains. RA8 may contribute to the dehiscence of anthers and microspore development in rice (Jeon et al. 1999). ZRP2 is a BURP protein in maize (*Zea mays*) that is expressed in the root cortex of parenchyma cells (Held et al. 1997). VfUSP is an abundant nonstorage seed protein found in the field bean, with unknown function. It is expressed during the early stages of zygotic embryogenesis, and the very early stages of in vitro embryogenesis (Bassuner et al. 1998). *ASG1* is specifically expressed during early embryo sac development in apomictic gynoecia, but is not expressed in the sexual gynoecia of *Panicum* spp. (Chen et al. 2005). *AtUSPL1* expression has been detected in specific cellular compartments, including the Golgi cisternae, prevacuolar vesicles, dense vesicles, and protein storage vacuoles of the parenchyma cells of cotyledons; thus, it may be involved in seed development (Van Son et al. 2009). The transcription of *BNM2* in oilseed rape is induced within microspore‐derived embryos; yet, the corresponding protein is confined to the seeds and localized to protein storage vacuoles (Boutilier et al. 1994). GmRD22 is an apoplastic‐localized BURP protein that interacts with a cell wall peroxidase in soybean. The ectopic expression of GmRD22 in transgenic thale cress and rice enhances lignin production under salinity stress (Wang et al. 2012). The cotton AtRD22‐like 1 gene *GhRDL1* is predominantly expressed in elongating fiber cells. This gene interacts with *α*‐expansin, which functions in wall loosening. The cooperation of these two proteins promotes plant growth and fruit production (Xu et al. 2013).

Several members of the BURP domain‐containing gene family have been reported to respond to stress treatments. Both SALI3‐2 and SALI5‐4a, two soybean BURP proteins, are induced by aluminum stress (Ragland and Soliman 1997). The BURP domain of SALI3‐2 may also be important in soybean tolerance to salt (Tang et al. 2007). AtRD22 is a drought‐responsive protein in thale cress, with its induction being mediated by ABA signaling. AtRD22 is often used as a reference for drought stress treatment in different plants. Protein biosynthesis for ABA‐dependent gene expression is required in the AtRD22 drought response (Yamaguchi‐Shinozaki and Shinozaki 1993b). The cooperation of RD22BP1 and AtMYB2 proteins as transcription factors induces the expression of the RD22 gene (Abe et al. 1997, 2003). The protein products of AtRD22 and AtUSPL1, both members of the thale cress BURP domain‐containing gene family, suppress drought stress response (Harshavardhan et al. 2014). ADR6 is a soybean BURP protein that is down‐regulated by auxin (Datta et al. 1993). *BnBDC1* is a shoot‐specific gene in oilseed rape that is down‐regulated by salicylic acid and UV irradiation and up‐regulated by ABA, NaCl, and mannitol (Yu et al. 2004). Fifteen of the 17 BURP genes identified from rice (excluding *OsBURP01* and *OsBURP13*) are induced by at least one of several stresses, including drought, salt, cold, and ABA treatment (Ding et al. 2009). The soybean genome contains 23 members of the BURP domain‐containing gene family, 17 of which are responsive to stress (Xu et al. 2010). Seven BURP genes from maize are upregulated by ABA and downregulated by cold. Two of these genes were both up‐ and downregulated by NaCl (Gan et al. 2011). Therefore, BURP family genes may be important for stress responses and adaptation, in addition to plant development.

The BURP domain‐containing gene family has not been studied for all plant lineages. However, the recent completion of sequencing and assembly of the BURP domain‐containing gene family provides an opportunity to understand the evolution of this family at the whole‐genome level. Thus, a comprehensive comparative genome study would help improve our understanding about the evolution and function of the BURP protein family. In this study, all BURP protein‐encoding sequence members from 13 plant species representing the major plant lineages were identified using available genome sequences. Phylogenetic, exon/intron structure, and motif analyses were conducted to trace the evolutionary history of the BURP domain‐containing gene family in plants. Rates of synonymous substitution (Ks) were also calculated to date duplication events. Functional divergence and adaptive evolution were analyzed at the amino acid level to examine the evolutionary drivers of BURP domain‐containing gene family function. The results presented in this study are expected to facilitate further research, by providing new insights about the evolutionary history of members of the BURP domain‐containing gene family.

## Materials and Methods

### Identification of BURP domain‐containing genes

BURP domain‐containing genes were collected from 15 fully sequenced genomes representing six plant lineages. Specifically, unicellular green algae *Chlamydomonas reinhardtii* (http://www.phytozome.org); multicellular green algae *Volvox carteri* (http://www.phytozome.org); moss *Physcomitrella patens* (http://genome.jgi-psf.org/); lycophyte *Selaginella moellendorffii* (http://genome.jgi-psf.org/); monocotyledonous angiosperms *Brachypodium distachyon* (http://www.brachypodium.org), *Setaria italica* (http://www.phytozome.org), *O. sativa* (http://rapdb.dna.affrc.go.jp/), *Sorghum bicolor* (http://genome.jgi-psf.org/), *Z. mays* (http://www.maize-sequence.org); and dicotyledonous angiosperms *A. thaliana* (http://www.arabidopsis.org/), *G. max* (http://genome.jgi-psf.org/soybean), *Cucumis sativus* (http://genome.jgi-psf.org/), *Citrus sinensis* (http://www.phytozome.org), *Brassica rapa* (http://brassicadb.org/brad/), and *Populus trichocarpa* (http://genome.jgi-psf.org/). Five gene sequences of the BURP domain‐containing gene family in *Arabidopsis* were downloaded from the Phytozome database (http://www.phytozome.org) and were separately blasted against corresponding plant genome annotation resources. Sequences were selected as candidate proteins if their E value was ≤1e‐10. The Simple Modular Architecture Research Tool (SMART; http://smart.embl-heidelberg.de/smart/batch.pl) and PFAM (http://pfam.sanger.ac.uk/) were used to confirm each predicted BURP domain‐containing protein sequence.

In our study, some identified BURP genes have more than one alternative splicing product. For further analysis, we selected the gene members that could translate the longest protein. All other alternative splicing members were regarded as redundant genes and were manually removed. Pseudogenes have different divergence characteristic to functional genes; thus, pseudogenes were excluded from our study. In general, genes that had incomplete open reading frames were regarded as pseudogenes and were excluded from our study.

### Alignment, phylogenetic, exon/intron structure motif, and promoter analyses

The multiple alignment of 125 full‐length protein sequences of BURP domain‐containing genes was performed using MUSCLE (Multiple Sequence Comparison by Log‐Expectation) (Edgar 2004a,b). The profiles of the created alignment protein sequences were used to construct an unrooted N‐J (neighbor‐joining) phylogenetic tree by MEGA6.0 (Tamura et al. 2013). ME (Minimum‐evolution) and ML (maximum‐likelihood) methods of phylogenetic inference were also used to construct phylogenetic trees to confirm tree topologies. The reliability of the interior branches of the phylogenetic trees was assessed through 1000‐iteration bootstrap resampling (Tamura et al. 2013). The online Gene Structure Display Server (GSDS: http://gsds.cbi.pku.edu.cn/) was used to explore the exon/intron structure of coding and genomic sequences (Guo et al. 2007). The motifs in the candidate BURP protein sequences were obtained using the MEME program (http://meme.sdsc.edu) (Bailey et al. 2009). MEME was run locally with the following parameters: number of repetitions = any number of repetitions, maximum number of motifs = 14, and optimum motif width was constrained to between 6 and 50 residues. The cis‐acting elements that regulate gene expression are distributed at 300–3000 bp upstream of the coding region. Therefore, the 1500 bp upstream of the coding region was selected as the promoter sequence, downloaded from Phytozome (http://www.phytozome.net), and submitted to PlantCARE (http://bioinformatics.psb.ugent.be/webtools/plantcare/html/) for analysis (Lescot et al. 2002).

### Dating the duplication events

Synonymous substitution rates (Ks) and their corresponding duplicated gene pairs were directly obtained from the Plant Genome Duplication Database (http://chibba.agtec.uga.edu/duplication/) (Tang et al. 2008). To avoid the risk of saturation within a 100‐kb range, anchors with Ks values greater than 1.0 were discarded. Duplicated gene pairs with fewer than three anchor points were also discarded. The Ks values of duplicated genes are expected to be similar over time. Therefore, Ks values were used as proxies for dates of segmental duplication events. Approximate dates of duplication events were derived using mean Ks values calculated from T = Ks/2*λ* (Yin et al. 2013), assuming clocklike rates of synonymous substitution (*λ*) of 6.1 × 10^−9^ for soybean (Blanc and Wolfe 2004), 1.4 × 10^−8^ for *Brassica* sp. (Wang et al. 2011), and 1.5 × 10^−8^ for *Arabidopsis* sp. (Bowers et al. 2003).

To assess the genetic distance between tandem‐duplicated pairs, fourfold synonymous third‐codon transversion (D4DTv) distance was calculated. The protein sequences of gene pairs were aligned using the program MUSCLE, and corresponding codon alignments were created using the online program PAL2NAL (http://www.bork.embl.de/pal2nal/) (Suyama et al. 2006). Corresponding codons were extracted from these alignments and were used to calculate the D4DTv distance between the members of each aligned pair. 4DTv is the transversion rate at fourfold synonymous codon positions, and ranged from zero (for recently duplicated paralogs) to ~0.5 (for paralogs derived from an ancient evolutionary past) (Liu and Zhu 2010).

### Tests of positive selection

To determine whether members of the BURP domain‐containing gene family underwent positive selection during evolution, a maximum‐likelihood approach was employed, using site and branch‐site models in the CODEML program of PAML v4.4 (Anisimova et al. 2002; Wong et al. 2004; Yang 2007). For the site models, two pairs of models were compared in PAML to test for heterogeneous selective pressures at codon sites. First, models M0 and M3 were compared using a test for heterogeneity between codon sites with respect to their *ω* ratios. Second, M7 and M8 were compared in a highly stringent test of positive selection. In parallel, a LRT (likelihood ratio test) was employed to compare these two models. When LRT suggested positive selection, Bayes Empirical Bayes analysis (BEB) was used to compute the posterior probability of each codon from the site class of positive selection under models M3 and M8. The branch‐site model assumes that all phylogenetic tree branches are divided a priori into foreground and background lineages and that the *ω* ratio varies between codon sites. Four site classes are present in the sequence: (1) with a small *ω* ratio (*ω*_0_), which is highly conserved in all lineages, (2) with neutral or weakly constrained sites, in which *ω * =  *ω*_1_, where *ω*_1_ is similar to or less than one, (3) and (4) with background lineages of *ω*_0_ or *ω*_1_, respectively, but with foreground branches of *ω*_2_ that may be greater than one. When constructing the LRTs, the null hypothesis fixes *ω*_2_ at one, allowing sites evolving under negative selection in the background lineages to be released from constraints, and to evolve neutrally in the foreground lineage. The alternative hypothesis constrains *ω*_2_ to be greater than or equal to one. The posterior probability (Qk) was determined, using the BEB method (Yang et al. 2005).

### Estimation of functional divergence

Type I functional divergence and type II functional divergence between the gene clusters of the BURP family were estimated through posterior analysis using the DIVERGE v2.0 program (Gaucher et al. 2002; Gu 1999, 2006). Functional type I divergence designates amino acid configurations that are highly conserved in cluster 1, but highly variable in cluster 2, and vice versa, implying that these residues have experienced altered functional constraints (Gu 2001). Type II divergence designates highly conserved amino acid configurations in the two gene clusters, but with very different biochemical properties. This phenomenon implies that the residues that belong to these configurations are responsible for functional specification (Lichtarge et al. 1996). The coefficients of type I and type II functional divergence (*θ*_I_ and *θ*_II_) between members of all pairs of interesting clusters were calculated. Values of *θ*_I_ and *θ*_II_ that were significantly greater than 0 implied site‐specific altered selective constraints or radical shifts in amino acid physiochemical properties following gene duplication and/or speciation (Lichtarge et al. 1996; Gaucher et al. 2002). Large Qk values indicate a high probability that evolutionary rates, or site‐level physiochemical amino acid properties, differ between two clusters (Gaucher et al. 2002).

## Results

### Identification of BURP domain‐containing genes

Fifteen plant species, representing six major plant lineages, were examined using the Phytozome database (http://www.phytozome.com). The six lineages included unicellular green algae *C*. *reinhardtii*, multicellular green algae *V. carteri*, moss *P. patens*, lycophyte *S. moellendorffii*, monocotyledonous angiosperms *B. distachyon, S. italica*,* O. sativa*,* S. bicolor*, and *Z. mays*, and dicotyledonous angiosperms *A. thaliana*,* G. max*,* C. sativus*,* C. sinensis*,* B. rapa*, and *P. trichocarpa*. The BLASTP search results identified 141 BURP domain‐containing homologue genes in the study species, excluding unicellular and multicellular green algae. Both PFAM and SMART databases confirmed the presence of the conserved domain in the BURP domain‐containing gene family. Sixteen candidate BURP domain‐containing gene sequences were found to have incomplete BURP domains and were excluded from our study. Of the 13 studied species (excluding algae), 125 BURP domain‐containing homologue genes were identified. Protein (Data S1), coding (Data S2), genomic (Data S3), and 1500‐bp nucleotide sequences upstream of the translation initiation codon (Data S4) were obtained from Phytozome.

### Phylogenetic relationships in the BURP domain‐containing gene family

**Figure 1:**
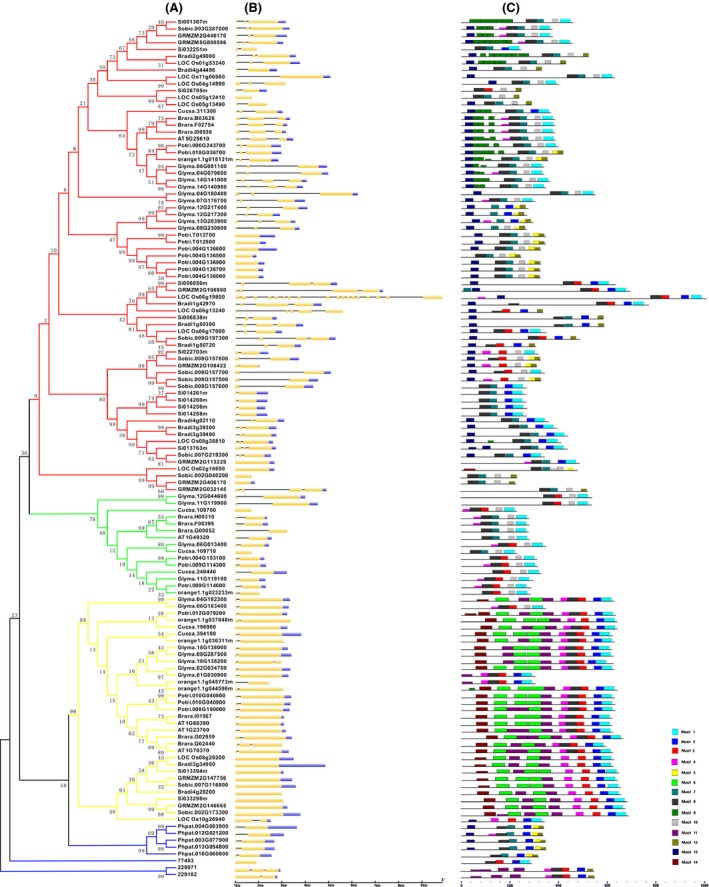
Phylogenetic relationships, exon/intron structure, and motif structures of BURP domain‐containing genes. (A) The neighbor‐joining phylogenetic tree was constructed based on a complete protein sequence alignment of 125 BURP domain‐containing genes, which were identified using MUSCLE and MEGA6. Numbers at the nodes represent bootstrap support (1000 iterations). The color of subclades indicates the four corresponding gene subfamilies. Genes with similar functions clustered together are indicated by filled blue circles; (B) Exon/intron structures of the BURP domain‐containing genes. Yellow boxes: exons; blue boxes: 3ʹ untranslated region; lines: introns. Box and line lengths are scaled based on gene length; (C) MEME motif search results. Conserved motifs are indicated in numbered, colored boxes.

The multiple alignment of 125 full‐length protein sequences of BURP domain‐containing genes (Data S5) was performed using MUSCLE (Multiple Sequence Comparison by Log‐Expectation), which has several additional advantages over other software used to create multiple alignment profiles of protein sequences (Edgar 2004a,b). To uncover the phylogenetic relationships within the BURP domain‐containing gene family, the alignment protein sequence profiles created here were used to construct an unrooted N‐J phylogenetic tree (Fig. 1) using MEGA6.0 (Tamura et al. 2013). Minimum‐evolution (ME) and maximum‐likelihood (ML) methods of phylogenetic inference were also used to construct phylogenetic trees to confirm tree topologies. ME and ML phylogenetic trees had similar topologies to the N‐J tree, with minor differences (Figs. S1, S2).

The N‐J tree was used in the subsequent steps. Based on the topology of the N‐J phylogenetic tree and the modular construction of BURP domain‐containing genes, the BURP family may be divided into four major subfamilies: PG1*β*‐like, BNM2‐like, BURPIII, and BURPIV (Fig. 1A). Of these four subfamilies, two (PG1*β*‐ and BNM2‐like) have already been reported (Granger et al. 2002). However, this study is the first to define the other two families, BURPIII and BURPIV. For the functional divergence and positive selection analyses, the previously reported USP‐ and RD22‐like subfamilies were considered subgroups of the BURPIII subfamily described in this study. While all of the members of the BURPIV subfamily are exclusively found in lower land plants (i.e., lycophytes and mosses), the other three subfamilies are found in higher land plants. In addition, while the PG1*β*‐like and BURPIII subfamilies are both found in dicotyledonous (hereafter dicot) and monocotyledonous (hereafter monocot) species, the BNM2‐like subfamily is only found in dicot species.

The online Gene Structure Display Server (Guo et al. 2007) was used to examine diverse exon/intron structures of all BURP domain‐containing genes. The results showed that three subfamilies (BURPIV, PG1*β*‐like, and BNM2‐like) have similar exon/intron structures, consisting mainly of two exons, while a few members had one or three exons (Fig. 1B). In contrast, the exon/intron structures of the BURPIII subfamily differed to those of the other three subfamilies. Of the 69 members of the BURPIII subfamily, approximately 45, 22, 16, and 13% had three, two, one, and four exons, respectively. The remaining 4% (3 members) had more than four exons; specifically, LOC_Os06 g13240, LOC_Os02 g18690, and LOC_Os06 g19800 had five, six, and 18 exons, respectively.

The motif detection software MEME (Multiple Em for Motif Elicitation) was used to investigate possible mechanisms of the structural evolution of BURP domain‐containing genes (Fig. 1C) (Bailey et al. 2009). The results showed the conservation of 14 motifs (Data S6). Proteins of the same subfamily had similar motif distributions (including type, order, and number of motifs), while those from different subfamilies had different motif distribution; in some cases, even proteins from the same subfamily had different motifs. Motifs 6, 11, and 14 were exclusively found in all proteins of the PG1*β*‐like subfamily, with the exception of orange1.1g045773m and LOC_Os10 g26940, which lacked motifs 6 and 14, and motifs 6, 11, and 14, respectively. Motif 9 was found in 72% of all proteins (18 of 25) of the RD22‐like subgroup, with 2–8 specific repeat units in one protein. Specific motifs may have important functions in the subfamily or subgroup.

All of the proteins from the PG1*β*‐like subfamily in the dicot species (excluding orange1.1g037848m) had motif 8, whereas this motif was absent in the proteins of the monocot species. Motif 8 was also found in all of the proteins of the other three subfamilies, except for seven protein sequences in the BURPIII subfamily; specifically, Glyma.12G217400, Glyma.12G217300, Glyma.13G283900, Glyma.08G230600, Si022703m, GRMZM2G108422, and Sobic.008G157500. These results indicate that motif 8 is present in members of lower plant species (BURPIV subfamily), but has been lost from members of the PG1*β*‐like subfamily in monocots. This divergence probably followed the monocot–dicot split, which occurred approximately 200 Mya. Motif 13 was identified from most proteins of the BURPIII and BURPIV subfamilies, but was not found in any members of the PG1*β*‐ or BNM2‐like subfamilies. Previous studies have reported that USP‐ and RD22‐like subfamilies (referring to USP‐ and RD22‐like subgroups of the BURP family) have short conserved segments located next to the hydrophobic, N‐terminal ends of BURP domain proteins (Granger et al. 2002). Our comparison of motif 13 with the conserved segment reported from previous studies suggests that they are the same, particularly with respect to amino acid composition and location in the protein sequence.

### Promoter analysis of BURP domain‐containing gene family

Quantitative real‐time analysis of transcript levels by Xu et al. (2010) showed that 15 of 23 soybean BURP genes had no expression specificity. Of the remaining eight genes, seven were specifically expressed in some of the tissues and one was not expressed in any of the studied tissues and organs. Stress treatment results also showed that 17 of the 23 soybean BURP genes were stress‐responsive, while the remaining six were not. Previous studies on rice, *P. trichocarpa*, maize, and sorghum also indicated that different members of the BURP domain‐containing gene family are expressed differently in various tissues or under different stress conditions (Shao et al., 2011; Ding et al. 2009; Gan et al. 2011). This difference may be linked to the divergence of the promoter regions of BURP domain‐containing genes. Promoters in the upstream region of genes are important for the developmental and/or environmental regulation of gene expression (Xue et al. 2008). Thus, profiles of cis‐acting elements may provide useful information about the regulatory mechanisms of gene expression.

In the current study, we used a computational tool, PlantCARE (Lescot et al. 2002) to identify cis‐acting elements in the 1500‐bp DNA sequence upstream of the translation initiation codon of BURP domain‐containing genes. We found that four types of elements are abundant in the promoter region of BURP domain‐containing genes (Table 1 and Data S7). As shown in the Table 1, the first class of cis‐acting elements contains plant hormone‐responsive elements and was concentrated in the promoter region of BURP genes. Examples of these elements included the TCA‐element (Pastuglia et al. 1997), TGA‐element (Guilfoyle et al. 1993), GARE‐motif (Ogawa et al. 2003), and ABRE (cis‐acting element involved in the abscisic acid responsiveness). ABRE appeared to be one of the most abundant hormone‐related cis‐acting elements in BURP domain‐containing genes (73 of 125; Data S7). This observation indicates that ABA regulates the expression of proteins in the BURP domain‐containing gene family. The abundance of the TGACG‐motif, GARE‐motif, and TCA‐element in BURP domain‐containing genes indicates that MeJA, gibberellin, and salicylic acid also help regulate BURP gene expression. Other elements, such as AuxRR‐core (Ballas et al. 1995), TGA‐box (Pascuzzi et al. 1998), motif IIb, P‐box (Kim et al. 1992), and TATC‐box (Jacobsen and Gu 1995), are also associated with auxin, ABA, and gibberellin responsiveness.

**Table 1:** Promoter analysis of BURP domain‐containing gene family

	Hormone‐responsive elements					Environmental stress‐related elements			
	TCA‐element^a^	TGA‐element	GARE‐motif	ABRE	TGACG‐motif	ARE	MBS	HSE	TC‐rich elements
PG1*β*	25/33	8/33	22/33	31/33	18/33	43/33	33/33	29/33	42/33
BNM2	11/15	2/15	9/15	10/15	12/15	22/15	24/15	22/15	22/15
BURPIII	45/69	18/69	40/69	119/69	102/69	88/69	117/69	51/69	69/69
BURPIV	3/8	4/8	4/8	6/8	8/8	19/8	11/8	4/8	4/8
Total^b^	84/125	32/125	75/125	166/125	140/125	172/125	185/125	106/125	137/125

a Total number of *cis*‐acting elements/members of BURP domain‐containing subfamilies;

b Total number of *cis*‐acting elements/members of BURP domain‐containing family.

The second class of abundant cis‐acting elements responds to external environment stresses. We observed that most BURP domain‐containing genes contained ARE (Klotz and Lagrimini 1996), MBS (Yamaguchi‐Shinozaki and Shinozaki 1993a), HSE (Freitas and Bertolini 2004), and TC‐rich elements (Klotz and Lagrimini 1996). ARE is associated with anaerobic induction. Therefore, the anaerobic regulation of BURP gene expression may depend on tissue type or developmental stage. The drought‐responsive element MBS is also abundant in the promoter region, with a mean number of 1.488 copies (Data S7 and Table 1). HSE‐ and TC‐rich repeats in BURP genes were abundant; thus, these genes may be involved in the plant response to heat stress, defense, and stress responsiveness. Other cis‐acting elements that respond to external environmental stresses were also found, including LTR, GC‐motif, box‐W1, W‐box, and WUN‐motif. This result indicates that members of plant‐specific BURP families are involved in plant responses to low‐temperature, anoxic‐specific inducibility, fungal elicitors, wounding, and pathogens.

Circadian elements are involved in circadian control (Pichersky et al. 1985) and represent the third most abundant class of cis‐acting elements in the promoter region of BURP domain‐containing genes (Data S7). The fourth most abundant type of element in the promoter region includes the G‐box (Sommer and Saedler 1986; Menkens et al. 1995), Box 4 (Lois et al. 1989), and Box I (Arguello‐Astorga and Herrera‐Estrella 1996), which are light‐responsive elements. The presence of diverse cis‐acting elements in the upstream regions of BURP domain‐containing genes indicates that this gene family has a wide range of functions, particularly with respect to plant responses to external environmental stress and hormone regulation.

### Duplication events in the BURP domain‐containing gene family

The three principal types of gene duplication that provide large amounts of genetic material for selection processes, and, therefore, evolution, are segmental duplication, tandem duplication, and transposition events, including replicative transposition and retroposition (Kong et al. 2007). In this study, we focused on segmental and tandem duplications. Our results show that 12.8% of BURP domain‐containing genes (16 of 125 genes) are segmentally duplicated (Table 2). This result indicates that segmental duplication has contributed to the expansion of the BURP domain‐containing gene family, particularly for plant species like *G. max*,* B. rapa*, and *A. thaliana*, which are the only species that contain segmental duplication gene pairs.

**Table 2:** Estimates of the dates of the segmental duplication events of the BURP domain‐containing gene family

Gene pairs		KS (mean ± SD)	Estimated time (mya)	GWD (mya)
AT1G70370	AT1G23760	0.795 ± 0.122	26.5	28–48
Brara.I00536	Brara.B03626	0.480 ± 0.203	17.1	13–17
Brara.F02754	Brara.I00536	0.451 ± 0.123	16.1	
Brara.F02754	Brara.B03626	0.441 ± 0.191	15.8	
Brara.G02440	Brara.G02959	0.380 ± 0.074	13.6	
Glyma.06G013400	Glyma.11G119100	0.553 ± 0.102	45.3	13,59
Glyma.06G013400	Glyma.12G044600	0.548 ± 0.163	44.9	
Glyma.12G217300	Glyma.13G283900	0.145 ± 0.083	11.9	
Glyma.02G034700	Glyma.01G030900	0.151 ± 0.051	12.3	
Glyma.06G081100	Glyma.04G079600	0.132 ± 0.042	10.8	

Tandem duplication events often generate multiple members of one family within the same or neighboring intergenic regions. In this study, we defined tandem‐duplicated genes as adjacent homologous genes on a single chromosome, with no more than 10 intervening genes between them (Ramamoorthy et al. 2008). Thirty two of the 125 BURP domain‐containing genes (25.6%) were derived from tandem duplication events (Table 3), but were only found in *G. max*,* P. trichocarpa*,* C. sinensis*,* C. sativus*,* S. italica*, and *S. bicolor*. This result indicates that tandem duplication has also contributed to the expansion of the BURP domain‐containing gene family. Furthermore, both segmental and tandem duplications were identified in BURPIII, PG1*β*‐like, and BNM2‐like subfamilies. This result indicates that both types of duplications have contributed to the expansion of these three subfamilies.

**Table 3:** Genes involved in tandem duplication and their 4DTv values

Species	Gene pairs		4_DTv_ value
Setaria italica	Si014261m.g	Si014260m.g	0.0000
	Si014261m.g	Si014258m.g	0.0270
	Si014260m.g	Si014258m.g	0.0268
	Si006838m.g	Si006050m.g	1.6000
Sorghum bicolor	Sobic.008G157800	Sobic.008G157700	0.8424
	Sobic.008G157800	Sobic.008G157500	0.4927
	Sobic.008G157800	Sobic.008G157600	0.5364
	Sobic.008G157700	Sobic.008G157500	0.6103
	Sobic.008G157700	Sobic.008G157600	0.5243
	Sobic.008G157500	Sobic.008G157600	0.1370
Citrus sinensis	Orange1.1g044596m	Orange1.1g036311m	0.6667
Glycine max	Glyma.14G140900	Glyma.14G141000	0.1259
	Glyma.11G119100	Glyma.11G119900	0.7027
	Glyma.12G217300	Glyma.12G217400	0.0504
	Glyma.18G138000	Glyma.18G138200	0.2310
Populus trichocarpa	Potri.006G243600	Potri.006G243700	0.0697
	Potri.T012700	Potri.T012500	0.0258
	Potri.004G136900	Potri.004G136500	0.0198
	Potri.004G136900	Potri.004G136700	0.0001
	Potri.004G136900	Potri.004G136000	0.0380
	Potri.004G136900	Potri.004G136600	0.0060
	Potri.004G136500	Potri.004G136700	0.0202
	Potri.004G136500	Potri.004G136000	0.0234
	Potri.004G136500	Potri.004G136600	0.0225
	Potri.004G136700	Potri.004G136000	0.0029
	Potri.004G136700	Potri.004G136600	0.0057
	Potri.004G136000	Potri.004G136600	0.0057
	Potri.009G114300	Potri.009G114600	0.5909
	Potri.010G040800	Potri.010G040900	0.0000
Cucumis sativus	Cucsa.109710	Cucsa.109700	0.9248

Some genes were identified in different segmental or tandem duplication pairs. Consequently, 16 identified segment duplication genes formed 10 pairs of segmental duplication genes, while 32 identified duplication genes formed 30 pairs of tandem duplication genes. By estimating the approximate ages of the segmental duplication events, we identified 10 pairs of segmental duplication genes in the BURP domain‐containing gene family (Table 2), when using on synonymous base substitution rates (Ks values). One pair of genes in *A. thaliana* (*AT1G70370* ¦ *AT1G23760*) was predicted to have diverged between 28 and 48 Mya, when recent large‐scale duplications occurred (Ermolaeva et al. 2003). Four pairs of BURP domain‐containing genes from *B. rapa* originated between 13.6 and 17.1 Mya. This prediction is consistent with data suggesting recent large‐scale duplications between 13 and 17 Mya (Yang et al. 1999; Town et al. 2006). Previous studies on soybean have demonstrated that two large‐scale duplication events occurred approximately 59 and 13 Mya, respectively (Schlueter et al. 2004; Schmutz et al. 2010). In the five pairs of BURP domain‐containing genes in soybean, two of five pairs of paralogous genes were estimated to have originated 45.3 and 44.9 Mya, respectively, while the other three pairs originated 12.23, 11.9, and 11.78 Mya, respectively. These time estimates are roughly consistent with the period of the two duplication events. These results indicate that segmentally duplicated genes were retained after the WGD (whole‐genome duplication) events during the evolution of each species. The two genes of each duplicated pair belonged to the same subfamilies. This result indicates that these genes have high sequence similarity, and might have similar functions or might have produced minor functional divergence.

The 4DTv distance (D4DTv) of tandem‐duplicated gene pairs was calculated using the PAML software package (Yang 2007). D4DTv ranges from 0 (for recently duplicated peptides) to ~0.5 (for paralogs with an ancient evolutionary past) (Liu and Zhu 2010). Seventeen pairs of tandem‐duplicated genes had smaller D4DTv (<0.1). This result indicates that these gene pairs appeared in the recent evolutionary past (Table 3). In comparison, the D4DTv of the other 14 pairs of tandem‐duplicated genes exceeded 0. This result indicates that these gene pairs appeared in the ancient evolutionary past.

In summary, both segmental and tandem duplication have contributed to the expansion of the BURP domain‐containing gene family in certain plant species, such as soybean. Segmental duplication appears to have contributed to the expansion of BURP genes in *B. rapa* and *A. thaliana*. In comparison, tandem duplication probably contributed to the expansion of BURP genes in *P. trichocarpa*,* S. italica*,* S. bicolor*,* C. sinensis*, and *C. sativus*. The genes involved in segmental duplication were retained after WGD events.

### Functional divergence in the BURP domain‐containing gene family

To investigate whether amino acid substitutions in the BURP domain‐containing gene family caused adaptive functional diversification, type I functional divergence and type II functional divergence between gene clusters in the BURP family were estimated through posterior analysis using the DIVERGE v2.0 program (Gu 1999, 2006; Gaucher et al. 2002). Type I functional divergence is the evolutionary process resulting in site‐specific shifts in evolutionary rates following gene duplication. Type II functional divergence is the process resulting in site‐specific amino acid physiochemical property shifts. These methods have been extensively applied in the study of various gene families because that they are not sensitive to the saturation of synonymous sites (Liu and Zhu 2008; Liu et al. 2009; Wang et al. 2009). The estimate was based on the neighbor‐joining tree approach. The BURPIV subfamily protein members were excluded because they did not cluster together and, therefore, could not be analyzed using this method.

The results of the posterior analysis showed that the coefficients of type I functional divergence (*θ*_I_) (ranging from 0.230 to 0.618) varied significantly among the three BURP domain‐containing gene subfamilies (*P* < 0.05). The selective constraints that operate on different types of BURP family members, leading to subgroup‐specific functional evolution after diversification, are highly differentiated, site‐specific, and altered. The coefficients of type II functional divergence *θ*_II_ were lower than 0 and were associated with high standard error estimates. Moreover, these coefficients were similar between pairs of subfamilies, such as PG1*β*‐ and BNM2‐like, PG1*β*‐like and BURPIII, and BNM2‐like and BURPIII (Table 4).

**Table 4:** Functional divergence between subfamilies of the BURP domain‐containing gene family

Group I	Group II	Type II *θ*_I_ ± SE	LRT	Qk >0.95	Critical amino acid sites	Type II *θ*_II_ ± SE	Qk >0.95	Critical amino acid sites
PG1*β*‐like	BNM2‐like	0.618 ± 0.080	59.287	7	181D,184R,273L,279^a^R, 309^a^K,332T,343G	−0.113 ± 0.238	13	178^a^L, 179E, **257** a **Y**, 264S, 312V, 316Q, **317K**, 328A, 348A, 383P, 385T, 386H, 388V
PG1*β*‐like	BURP III	0.591 ± 0.071	69.307	7	184^a^R,248^a^A,255^a^E**,257** a **Y, 317K**,343G,391^a^S	−0.824 ± 0.520		None
BNM2‐like	BURP III	0.230 ± 0.058	15.684	1	273L	−2.010 ± 0.905		None

*θ*_I_ and *θ*_II_, the coefficients of type I and type II functional divergence; LRT, likelihood ratio statistic; Qk, posterior probability;

a Sites also responsible for the positive selection;

Sites in bold font indicate those responsible for both type I and type II functional divergence.

**Figure 2:**
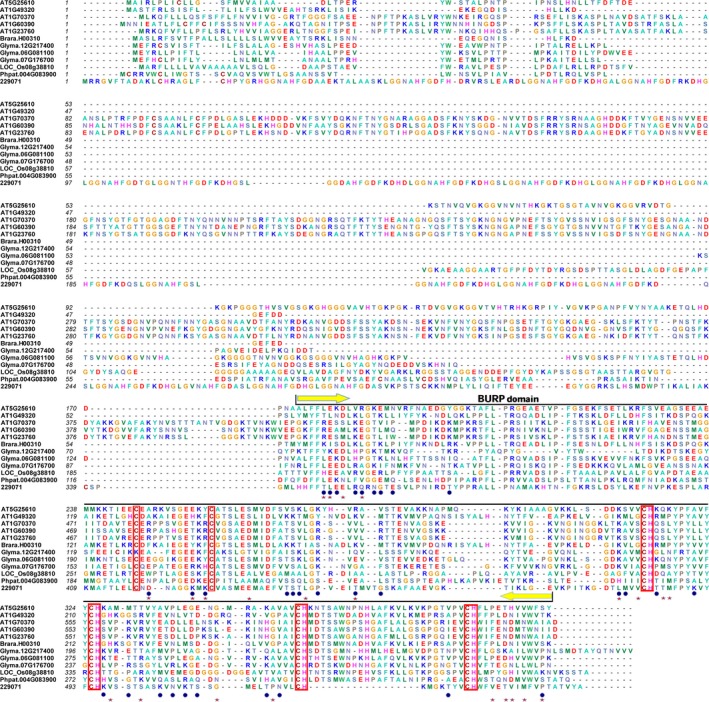
Multiple sequence alignment of several BURP domain‐containing protein sequences. The position of the BURP domain is indicated at the top of each sequence. The sites of two conserved cysteine (C) residues, and four repeats of the cysteine‐histidine motif, which are located in the C‐terminus BURP domain, are indicated by red frames. The critical amino acid sites of adaptive selection and functional divergence are marked by the red stars and blue circles, respectively.

The BEB method was used to determine the posterior probability of divergence (Qk) (Yang et al. 2005) for each amino acid site property. We aimed to identify critical amino acid sites that may be relevant to functional divergence between BURP domain‐containing gene subfamilies. Large Qk values indicate a high probability that evolutionary rates, or site‐level physiochemical amino acid properties, differ between two clusters (Gaucher et al. 2002). To reduce false positives, type I and type II functional divergence‐related residues between the BURP subfamilies (Table 4 and Fig. 2) with Qk > 0.95 were excluded from the study.

Critical amino acid sites with potential relevance to type I functional divergence were identified in the BURP domain‐containing gene family (amino acids referring to the AT5G25610 sequence). Only one amino acid site, potentially relevant to type I functional divergence, was identified between the BNM2‐like and BURPIII subfamilies. In contrast, seven amino acid sites were identified between PG1*β*‐ and BNM2‐like and between PG1*β*‐like and BURPIII subfamilies. This result indicates that an evolutionary shift occurred at these sites. Unexpectedly, only amino acid sites that were crucial for type II functional divergence between PG1*β*‐ and BNM2‐like subfamilies were identified. Thirteen such critical sites were identified. This result indicates that there has been a radical shift in amino acid properties. Furthermore, two amino acids, crucial for both type I and type II functional divergence, indicate that shifts in evolutionary rates and altered amino acid physicochemical properties co‐occurred at these amino acid sites. In addition, PG1*β*‐ and BNM2‐like subfamilies had relatively larger coefficients of type I and type II functional divergence, and several more sites that were relevant to functional divergence, than the PG1*β*‐like and BURPIII, and BNM2‐like and BURPIII subfamilies. Therefore, there may have been greater type I and type II functional divergence between the PG1*β*‐ and BNM2‐like subfamilies compared to that between PG1*β*‐like and BURPIII and between BNM2‐like and BURPIII subfamilies. A lower degree of functional divergence was detected between PG1*β*‐like and BURPIII and between BNM2‐like and BURPIII subfamilies. Therefore, the BNM2‐like and BURPIII subfamilies may have a closer phylogenetic relationship than BNM2‐ and PG1*β*‐like subfamilies.

### Positive selection in the BURP domain‐containing gene family

To determine whether members of the BURP domain‐containing gene family underwent positive selection during evolution, a maximum‐likelihood approach, using site and branch‐site models in the CODEML program of PAML v4.4, was employed (Anisimova et al. 2002; Wong et al. 2004; Yang 2007). The substitution rate ratio (*ω*) of nonsynonymous (dN) to synonymous (dS) mutations is an important indicator of positive selection at the molecular level and was calculated in our analysis. The dN/dS ratio is expected to be (1) 1 in the case of genes subjected to neutral selection, (2) <1 for genes subjected to negative selection, and (3) >1 for genes subjected to positive selection (Yang 2000a). For site models, LRTs were used on the codon site models M0, M3, M7, and M8, to test variational *ω* ratios at amino acid sites (Table 5). M0 was a one‐ratio model that assumed one *ω* ratio at all sites. M3 was a discrete model, in which the data were used to infer the probabilities of each site being subject to purifying, neutral, and positive selection (p0, p1, and p2, respectively), in addition to their corresponding unconstrained Ka/Ks ratios (*ω*_0_, *ω*_1_, and *ω*_2_). M7 was a null model, in which *ω* was assumed to have a beta distribution between 0 and 1. M8 combined the *β* and *ω* models and added one extra class with the same ratio *ω*_1_ (Yang 2000b). In our study, two pairs of models were contrasted in PAML, to test for heterogeneous selective pressures at codon sites.

**Table 5:** Tests for positive selection among codons of BURP domain‐containing genes using site models

Models	p^a^	Estimates of parameters	InL	2Δl	Positively selected sites^b^
M0 (one‐ratio)	1	*ω* = 0.16055	−20918.0		None
M3 (discrete)	5	p_0_ = 0.12267 p_1_ = 0.40583 p_2_ = 0.47150 *ω*_1_ = 0.00407 *ω*_2_ = 0.09450 *ω*_3_ = 0.28171	−20403.3	1029.4 (M3 vs. M0)	None
M7 (beta)	2	p = 0.79669 q = 4.17489	−20362.5		Not allowed
M8 (beta&ɯ)	4	p_0_ = 0.99999 p = 1.03218 q = 1.97229 (p_1_ = 0.00001) *ω* = 2.56995	−24885.1	9045.2 (M8 vs. M7)	**178** c **L, 180K**,** 183V**,** 184** c **R**,** 186K**,** 187E**,** 189N**,** 198Y**,** 248** c **A**,** 255** c **E**,** 257** c **Y**,**270V**, 272K, **275K**,** 279** c **R**,** 281V**,** 309** c **K**,** 310S**,** 315K**,** 321A**,** 327K**,** 330M**,** 335A**,** 337P**,** 339E**,** 341E**,** 347K**, 349V, 376V, **391** cS

a Number of parameters in the *ω* distribution.

b Positive selection sites are inferred at posterior probabilities >95% with those reaching 99% shown in bold.

c Sites also found to be involved in the functional divergence.

The first pair of models (M0 vs. M3), which tested for contrasting heterogeneous selective pressures at codon sites, yielded an LRT statistic of 1029.4 (*P* < 0.05). This result indicates that one category of *ω* was insufficient to describe variability in selection pressure across amino acid sites. In addition, this result indicates that the BURP domain‐containing gene family overcame strong selective pressure during evolution. The comparison of the second pair of models (M7 vs. M8) revealed that ~0.001% of codons fell within an estimated *ω* value of 2.56995 (which is an indicator of positive selection).

Thirty codon site candidates were identified from the M8 model using Bayesian posterior probabilities (Yang 1997) and included 27 positive selection sites with *P* < 0.01, and three sites with *P* < 0.05. The nine positive selection sites, identified from the site model, were also responsible for functional divergence. Six of these sites were responsible for type I functional divergence, two sites for type II functional divergence, and one site for both. Finally, when the BNM2‐like subfamily was defined as the foreground branch, branch‐site model analyses identified only two positive selection sites (Table 6). In contrast, no positive selection sites were found when the PG1*β*‐like, BURPIII, and BURPIV subfamilies were defined as foreground branches. This result indicates that the BNM2‐like subfamily is not considerably more conserved than the PG1*β*‐like, BURPIII, and BURPIV subfamilies.

**Table 6:** Parameter estimation and likelihood ratio tests for the branch‐site models

Cluster	Site class	Proportion	Background ɯ	Foreground ɯ	InL	2Δl	Positive selection sites^a^
PG1*β*‐like	0	0.86612	0.16585	0.16585	−20839.9	156.2 (M2 vs. M0)	None
	1	0.09642	1.00000	1.00000			
	2a	0.03370	0.16585	998.99217			
	2b	0.00375	1.00000	998.99217			
BNM2‐like	0	0.79685	0.16490	0.16490	−20836.0	164 (M2 vs. M0)	179^b^E, 271S
	1	0.08773	1.00000	1.00000			
	2a	0.10438	0.16490	62.88724			
	2b	0.01144	1.00000	62.88724			
BURP III	0	0.82963	0.16564	0.16564	−20839.3	157.4 (M2 vs. M0)	None
	1	0.09127	1.00000	1.00000			
	2a	0.07125	0.16564	999.00000			
	2b	0.00784	1.00000	999.00000			
BURP IV	0	0.81364	0.16582	0.16582	−20838.4	159.2 (M2 vs. M0)	None
	1	0.09005	1.00000	1.00000			
	2a	0.08671	0.16582	5.66019			
	2b	0.00960	1.00000	5.66109			

a Positive selection sites are inferred at posterior probabilities >95%.

b Sites also found to be involved in the functional divergence.

## Discussion

### Origin of the BURP domain‐containing gene family

Fifteen plant species, representing six major plant lineages, were examined using the Phytozome database. The BLASTP search results identified BURP domain‐containing homologue genes in all study species, except for unicellular and multicellular green algae. We failed to identify BURP domain‐containing genes in lower hydrobiotic algae, namely the chlorophytes *Coccomyxa subellipsoidea*,* Micromonas pusilla CCMP1545*,* M. pusilla RCC299*, and *Ostreococcus lucimarinus*. These negative results are consistent with the speculation of Xu et al. (Xu et al. 2010) that BURP family genes appeared when plants shifted from water to land, where the environment was more variable (Xu et al. 2010). This result supports the theory that this gene family is involved in plant adaptation to adverse and variable environments, such as terrestrial habitats.

The N‐J phylogenetic tree developed in our study included 125 distinct protein sequences and strongly indicated that these genes are separated into four subfamilies: PG1*β*‐like, BNM2‐like, BURPIII, and BURPIV (Fig. 1A). This classification was supported by the results of motif and exon/intron analyses. Of the four subfamilies, the BURPIV subfamily was only present in lower land plants (i.e., lycophytes and mosses), whereas the PG1*β*‐like, BNM2‐like, and BURPIII subfamilies were only present in higher land plants (i.e., angiosperms). In addition, the BNM2‐like subfamily was found exclusively in dicots. Overall, these results indicate that BURP domain‐containing genes shared a common ancestor before lower and higher land plants diverged. We propose an evolutionary trajectory for the BURP domain‐containing gene family based on the phylogenetic tree. Initially, all members of the BURP domain‐containing gene family shared a common ancestor. Subsequently, lower land plant members (belonging to the BURPIV subfamily) started to diverge. Then, the PG1*β*‐like subfamily, which contains both monocot and dicot members, diverged before the monocot–dicot split approximately 200 Mya. A similar divergence occurred for the BURPIII subfamily, which contains both monocot and dicot members. Finally, the BNM2‐like subfamily, which is exclusive to dicots, formed after the monocot–dicot split.

In the phylogenetic tree generated by our study, most of the closely related members had common motif composition. This result indicates that functional similarities exist among the BURP domain‐containing proteins within the same subfamily. These results also support the importance of using phylogenetic analysis in functional genomics studies. In the N‐J tree (Fig. 1A), three *B. rapa* BURP proteins (brara.H00310, brara.F00395, and brara.G00052) were well clustered with ATUSPL1 (AT1G49320), a protein that is involved in *A. thaliana* drought tolerance and seed development (Van Son et al. 2009; Harshavardhan et al. 2014). This clustering indicates that the three *B. rapa* BURP proteins and the ATUSPL1 protein have similar functions. Similar cases were also identified for other clusters; (1) ATRD22 (AT5G25610) ¦ brara.B03626 ¦ brara.F02754 ¦ brara.I00536, (2) OsRAFTIN (LOC_Os08 g38810) ¦ Bradi3 g39300 ¦ Bradi3 g39490 ¦ Sobic.007G219300 ¦ Si013763m ¦ GRMZM2G113229, (3) SALI3‐2 (Glyma.12G217400) ¦ Glyma.12G217300 ¦ Glyma.13G283900 ¦ Glyma.08G230600, (4) GmRD22 (Glyma.06G081100) ¦ Glyma.04G079600, and (5) SCB1(Glyma.07G176700) ¦ Glyma.04G180400. These proteins have a range of functions. For instance, ATRD22 is involved in *A. thaliana* seed development and drought tolerance (Yamaguchi‐Shinozaki and Shinozaki 1993b; Harshavardhan et al. 2014). OsRAFTIN transports sporopollenin from the tapetum to the developing microspores via Ubisch bodies (Wang et al. 2003). SALI3‐2 is a vacuole‐localized BURP‐domain protein that is thought to confer heavy metal tolerance to plants (Ragland and Soliman 1997; Tang et al. 2007). GmRD22 interacts with a cell wall peroxidase and affects cell wall integrity (Wang et al. 2012). SCB1 may contribute to the differentiation of seed coat cells (Batchelor et al. 2002).

### Expansion pattern of the BURP domain‐containing gene family

Genes involved in stress responses may have a high probability of retention following tandem duplication. Such tandem duplicates may be important for the adaptive evolution of plants to rapidly changing environments (Hanada et al. 2008). Several BURP domain‐containing genes have been identified as being involved in a variety of stress responses. For instance, we demonstrated that most BURP genes are involved in tandem duplication events. Specifically, 38.1% (8 of 21), 68.8% (11 of 16), 38.5% (5 of 13), 40% (4 of 10), 33.3% (2 of 6), and 33.3% (2 of 6) of BURP domain‐containing genes are involved in the stress responses of *G. max*,* P. trichocarpa*,* S. italica*,* S. bicolor*,* C. sinensis*, and *C. sativus*, respectively (Table 3). In addition, most pairs of tandem duplication genes in *S. bicolor*,* C. sinensis*,* G. max*,* P. trichocarpa*, and *C. sativus* had 4DTv values greater than 0. This result indicates that these gene pairs originated in the ancient evolutionary past, supporting the findings of Hanada et al. (Hanada et al. 2008).

Segmental duplication genes were identified in *G. max*,* B. rapa*, and *A. thaliana* (which were also retained by WGD), indicating that large‐scale duplication have contributed to the expansion of the BURP domain‐containing gene family (Table 2). In addition, BURP domain‐containing genes from different species did not share a common expansion model. Both tandem and segmental duplication had similar important roles in soybean. Only segmental duplications were identified in *B. rapa* and *A. thaliana*, whereas only tandem duplications were identified in *P. trichocarpa*,* S. italica*,* S. bicolor*,* C. sinensis*, and *C. sativus*. Of interest, the two genes that were derived from tandem duplication events and segmental duplication events belonged to the same subfamilies. This result indicates that these genes were not subject to evolutionary divergence after duplication. The estimated dates of origin of all deduced BURP domain‐containing paralogous gene pairs ranged from 45.3 to 11.9 Mya (Tables 2, 3). Thus, all deduced tandemly duplicated genes may have originated after the speciation of their respective species. Overall, our results clearly indicate that these BURP‐duplicated genes postdate the split between monocots and dicots, which is thought to have occurred approximately 200 Mya.

Overall, our results indicate that this gene family originated from a common ancestor, followed by lineage‐specific expansion and divergence in each lineage and species during evolution. Species‐specific expansion primarily occurred by tandem duplication in *G. max*,* P. trichocarpa*,* S. italica*,* S. bicolor*,* C. sinensis*, and *C. sativus* species and is likely to have contributed to the large size of the BURP domain‐containing gene family. Large‐scale duplication may have also been involved in the expansion of the BURP domain‐containing gene family for *G. max*,* B. rapa*, and *A. thaliana*.

### Function of the BURP domain

In general, a typical BURP‐domain protein consists of three or four modules, specifically, an N‐terminal hydrophobic domain (putative transit peptide), a short conserved segment or other short segment, an optional repeat domain (consisting of repeated units which are unique to each member), and a BURP domain at the C‐terminus (Hattori et al. 1998). Hattori et al. suggested that the BURP domain might be involved in targeting the attached polypeptide to, or immobilization at, a defined subcellular location, such as the cell wall. Thus, the repeated CH motif may provide an anchor for attachment to the cell wall by interacting with sulfated proteins or other nonproteinaceous sulfated compounds in the cell wall (Hattori et al. 1998). In the present study, all BURP proteins had four repeated CH motifs and two additional cysteine residues (Fig. 2), which had a well‐conserved distance. Xu et al. (2013) fused the BURP domain 125–335 aa of GhRDL1, which is a BURP gene in cotton (*Gossypium* sp.) with a YFP (yellow fluorescent protein) to generate transgenic *Arabidopsis* plants. The fluorescence signal generated by YFP was focused on the cell wall of the root cells (Xu et al. 2013). However, when an N‐terminal fragment (1–124 aa) was used instead of 125–335 aa, YFP fluorescence was spread throughout the protoplast, with no specific subcellular distribution. This result indicates that the BURP domain is involved in cell wall localization. Wang et al. (2012) demonstrated that the BURP domain of GmRD22 (Glyma.06G081100) is an indispensable determinative factor in subcellular localization. In addition, Tang et al. (2014) confirmed that the N‐terminal putative signal peptides of both SALI3‐2 (Glyma.12G217400) and AtRD22 (AT5G25610) are essential and critical domains for targeting proteins to their destinations.

PG1*β* forms a complex with the catalytic polygalacturonase isoenzyme, PG2, and may interact with the structural components of the cell wall, in addition to the PG2 catalytic subunit, to immobilize or regulate polygalacturonase‐enzyme complex activity (Zheng et al. 1992). The region mainly consists of repeated units, with two successive incisions in the BURP protein causing it to become the functional polygalacturonase *β*‐subunit (Zheng et al. 1992). The cotton AtRD22‐like 1 gene (GhRDL1) interacts with *α*‐expansin, and their co‐expression may promote plant growth and fruit production (Xu et al. 2013). The apoplastic GmRD22 (Glyma.06G081100) interacts with a cell wall peroxidase, with the ectopic expression of GmRD22 in *A*. *thaliana* and rice possibly increasing the lignin content of the cell wall under salinity stress (Wang et al. 2012). Both GhRDL1 and GmRD22 are members of the RD22‐like subgroup that contains approximately 30 aa conserved segments attached to the N‐terminal signal peptide, in addition to several copies of a repeated unit that is unique to each member of this subgroup. OsRAFTIN (LOC_Os08 g38810) transports sporopollenin from the tapetum to developing microspores via Ubisch bodies, and has a distinctive short segment behind the presumptive signal peptide, in addition to two copies of an exclusive repeated unit (Wang et al. 2003).

Based on previous function research about the different members of BURP domain‐containing gene family, we suggest that different BURP protein modules have diverse roles in protein function. First, N‐terminal hydrophobic segments, which are putative transit peptides, may be the critical domains for targeting proteins to their destinations. C‐terminal BURP domains may be involved in targeting the attached polypeptide to a defined subcellular location, such as the cell wall. Subsequently, segments, which occur between the N‐terminal hydrophobic segments and C‐terminal BURP domains, interact with various substrates, such as *α*‐Expansin and cell wall peroxidase, to realize specific protein functions.

### Functional divergence and positive selection analysis

Gene duplications are a primary driving force in the evolution of genomes and genetic systems (Moore and Puruggananm 2003). Amino acid site mutation is frequent, with the accumulation of mutations potentially contributing to the functional divergence of duplicated genes (Blanc and Wolfe 2004; Gu et al. 2005; Sémon and Wolfe 2007; Ha et al. 2009). Typically, an amino acid residue is highly conserved in one duplicate gene, but is highly variable in the other duplicate (Zheng et al. 2007). Thus, we estimated type I and type II functional divergence between gene clusters of the BURP family by posterior analysis using the DIVERGE v2.0 program (Table 4). The analysis showed that functional divergence mainly occurs between pairs of subfamilies (e.g., PG1*β*‐ and BNM2‐like or PG1*β*‐like and BURPIII). Thus, the function of PG1*β*‐like may diverge with respect to the two subfamilies, BNM2‐like and BURPIII. Through functional divergence analysis, critical amino acid sites were detected. These sites have made important contributions to the functional divergence among the four BURP domain‐containing gene subfamilies. In addition, functional divergence might reflect the existence of long‐term selective pressures.

We used both site models and branch‐site models to detect positive selection. The site models predicted that 30 sites have undergone positive selection (Table 5). This result indicates that the BURP domain‐containing gene family has experienced high positive selection pressure. In contrast to site models, branch‐site models (Table 6) revealed just two positive selection sites in the BNM2‐like subfamily, none of which were predicted to have undergone positive selection among the PG1*β*‐like, BURP III, or BURPIV subfamilies. Both site and branch‐site models showed that each subfamily seems to have been conserved in the evolutionary process, even though the BURP domain‐containing gene family is predicted to have undergone positive selection. Finally, nine sites were responsible for both functional divergence and positive selection (178L, 179E, 184R, 248A, 255E, 257Y, 279R, 309K, and 391S; Fig. 2). These sites were found to be important in the evolutionary history of the BURP domain‐containing gene family. Of interest, all of the sites responsible for functional divergence and positive selection were found in the BURP domain. This observation indicates that this domain is involved in targeting the attached polypeptide to a defined subcellular location. Both functional divergence and positive selection analysis confirmed that the BURP domain contributes to functional divergence and that the subcellular localization of BURP proteins may generate differentiation.

## Conclusions

In this study, 125 BURP protein‐encoding genes were identified from four main lineages that included 13 species of higher plants. No members of the BURP protein family were identified from unicellular and multicellular green algae, indicating that BURP genes appeared when plants started to move from aquatic to terrestrial environments, where stresses associated with environmental variability are greater. The appearance of BURP genes at this stage of plant evolution also implies that the BURP gene family may contribute to plant adaptation to adverse and variable environments, such as the land environments that they came to colonize around the time that this gene family started to appear in the genome. BURP protein family genes showed different expansion patterns in different species. Several plant‐responsive elements to both hormones and external environmental are abundant in the promoter region of BURP protein‐encoding genes, suggesting that BURP proteins have an important influence on the stress responses of plants. This finding is consistent with previous reports stating that BURP domain‐containing gene members influence plant responses to various stress treatments. Furthermore, significant site‐specific selective constraints may have acted on many BURP domain‐containing genes, after gene duplication, leading to subfamily‐specific functional evolution. This functional divergence may reflect long‐term selective pressures on the gene family. Finally, all of the critical amino acid sites for functional divergence and positive selection detected in our study were located in the C‐terminal BURP domain. The results of this study are expected to contribute toward improving our understanding about the complexity, function, and evolution of the BURP domain‐containing gene family in green plants.

## Conflict of Interest

The authors declare that they have no competing interests.

## Supporting information


**Figure S1.** Minimum evolution (ME) phylogenetic tree of the BURP domain‐containing gene family.

**Figure S2.** Maximum likelihood (ML) phylogenetic tree of BURP domain‐containing gene family.

**Data S1.** Protein sequence data for the BURP domain‐containing gene family.

**Data S2.** Coding sequence data for the BURP domain‐containing gene family.

**Data S3.** Genome sequence data for the BURP domain‐containing gene family.

**Data S4.** 1500 bp of nucleotide sequences upstream of the translation initiation codon of BURP genes.

**Data S5.** Multiple sequence alignment of BURP domain‐containing gene family.

**Data S6.** Schematic of motifs of BURP domain‐containing proteins. The schematic was derived from MEME. The order of motifs of the BURP domain‐containing proteins in the schematic was automatically generated by MEME according to scores.

**Data S7.** Promoter analysis performed on the BURP domain‐containing gene family. Locus names, cis‐acting element names, and mean number of different types of cis‐element copies are listed.
