# Supplementary figures and images for: The divergence and positive selection of the plant‐specific BURP‐containing protein family
Source: Ecol Evol. 2015 Nov 2;5(22):5394–412. doi: 10.1002/ece3.1792 (PMC6102523; doi:10.1002/ece3.1792)

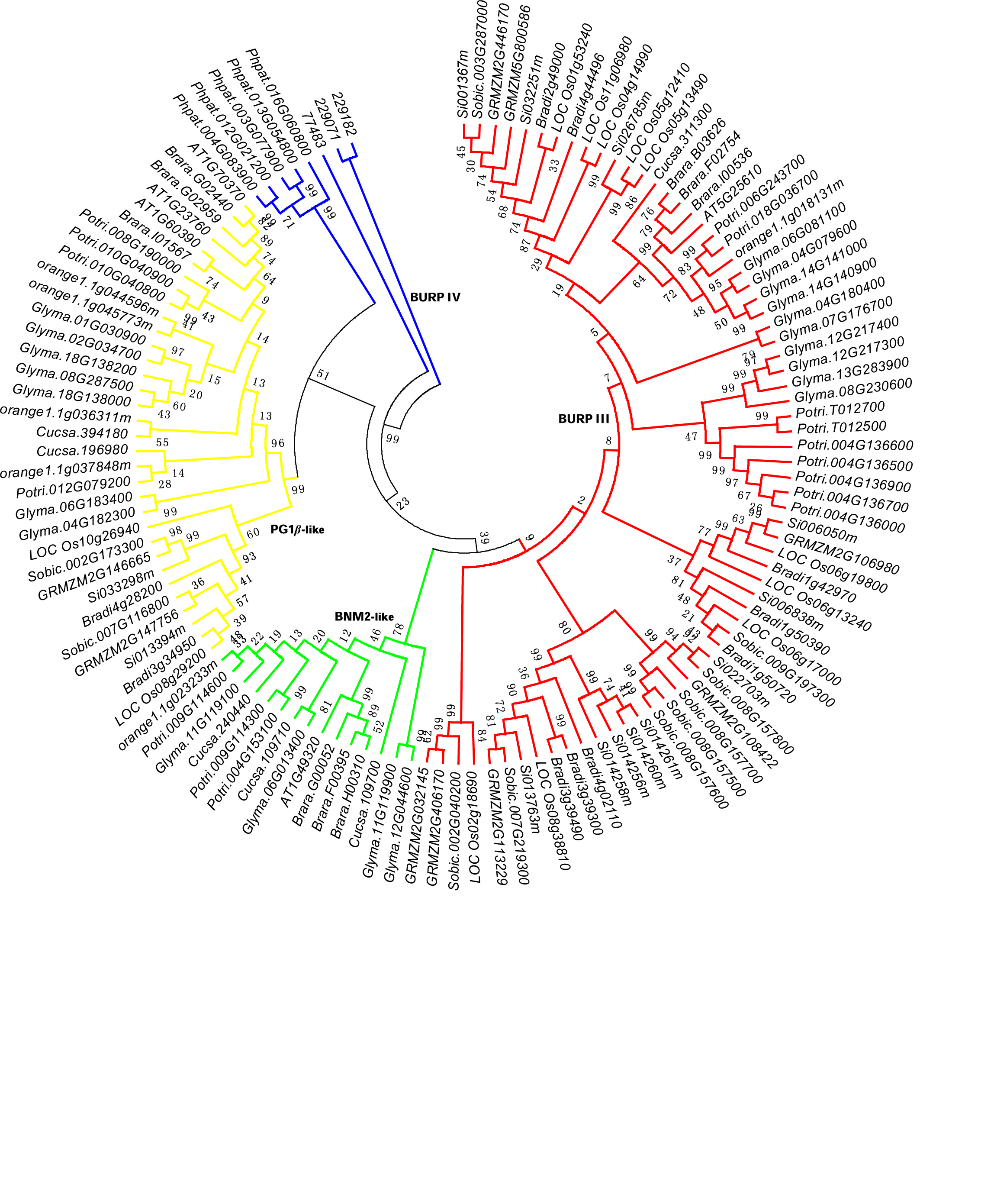

Supplement: Supplementary file 1 — Figure S1. Minimum evolution (ME) phylogenetic tree of the BURP domain‐containing gene family. [file ECE3-5-5394-s001.png]

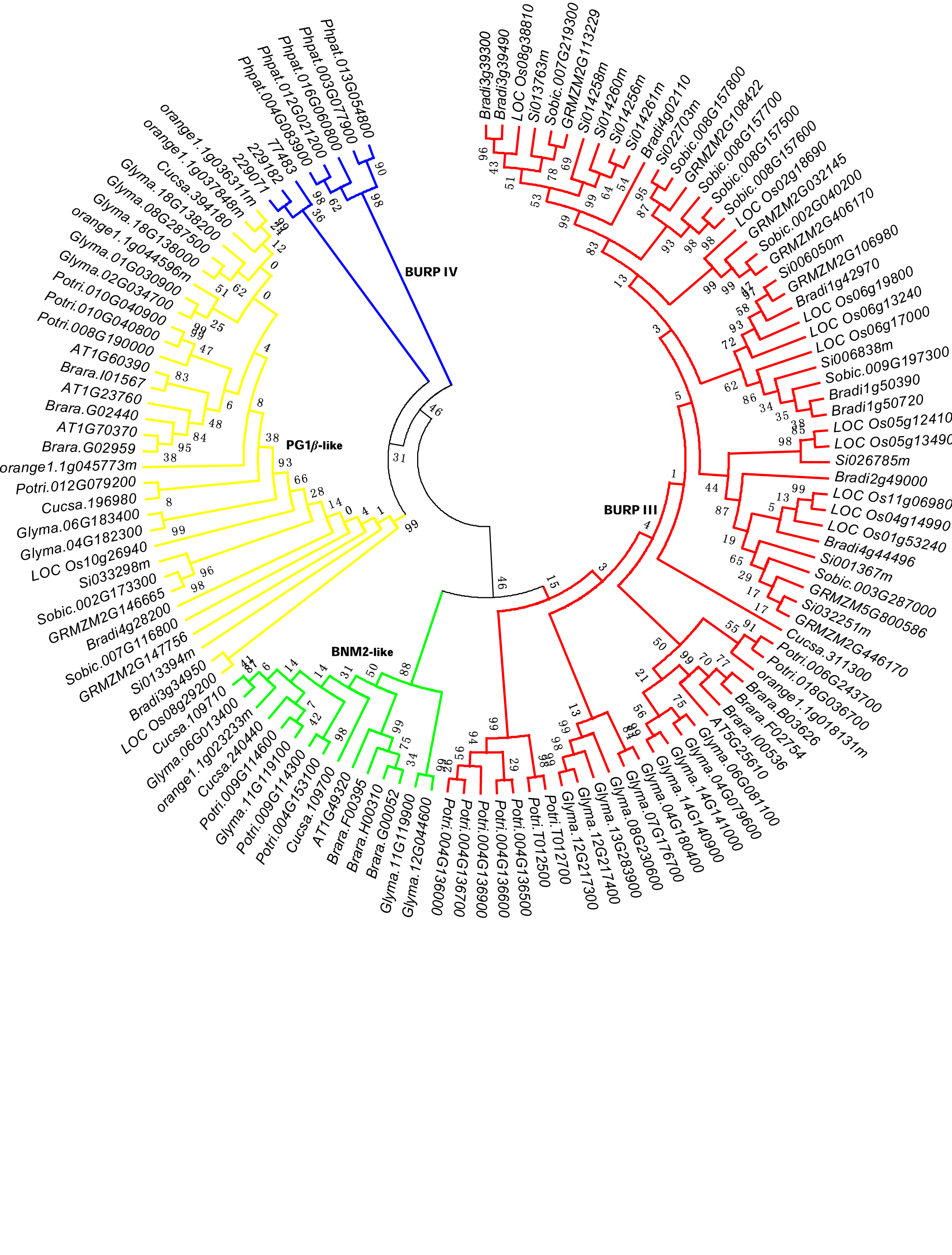

Supplement: Supplementary file 2 — Figure S2. Maximum likelihood (ML) phylogenetic tree of BURP domain‐containing gene family. [file ECE3-5-5394-s002.png]
