# Supplementary material for: The divergence and positive selection of the plant‐specific BURP‐containing protein family
Source: Ecol Evol. 2015 Nov 2;5(22):5394–412. doi: 10.1002/ece3.1792 (PMC6102523; doi:10.1002/ece3.1792)

**Data S6:** Schematic diagram of motifs of BURP domain-containing proteins.

The schematic diagram was derived from MEME. The order of motifs of the BURP domain-containing proteins in the diagram was automatically generated by MEME according to scores.


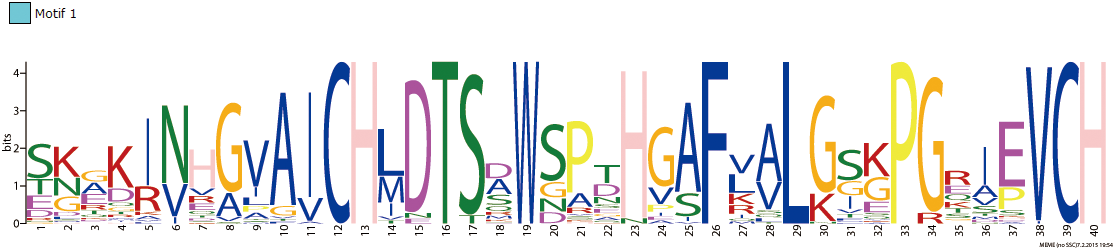


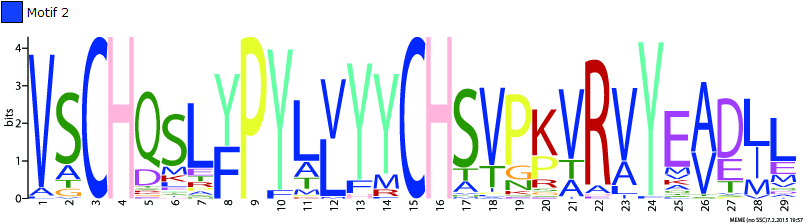


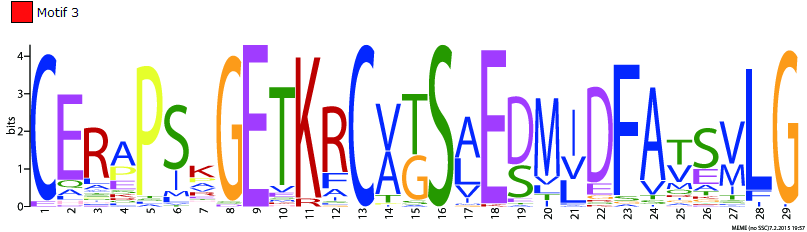


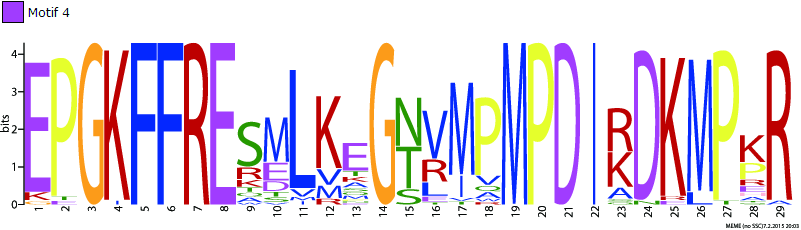


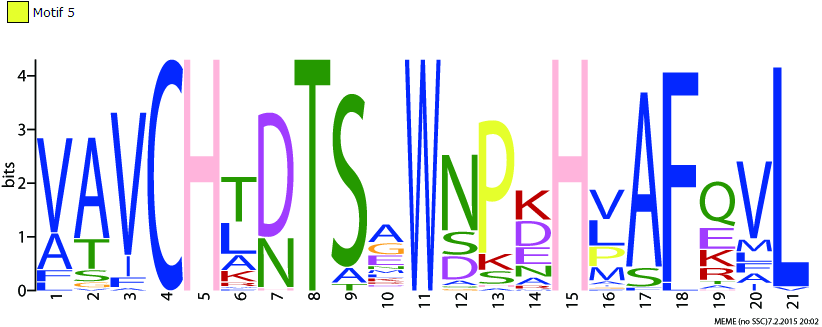


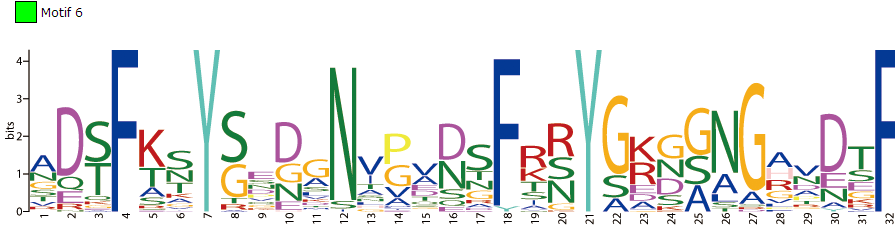


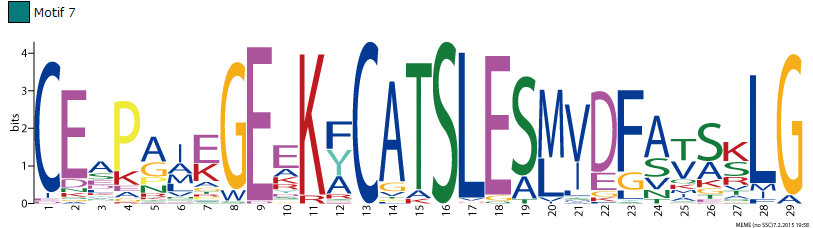


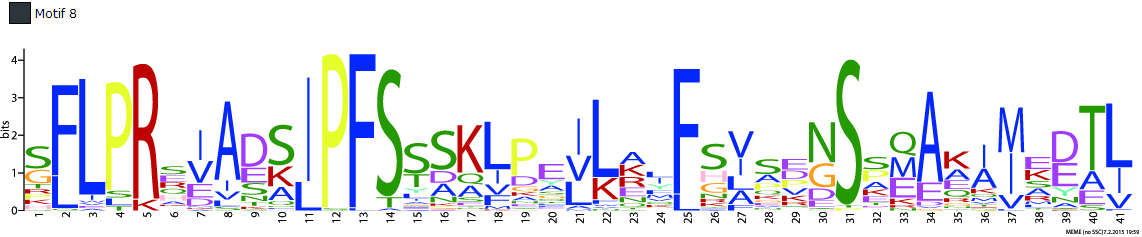


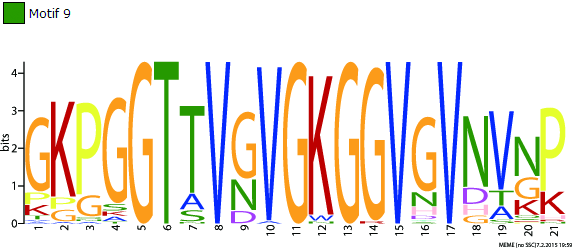


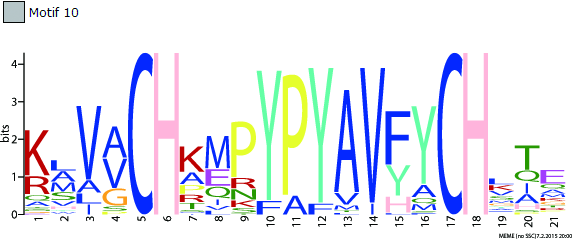


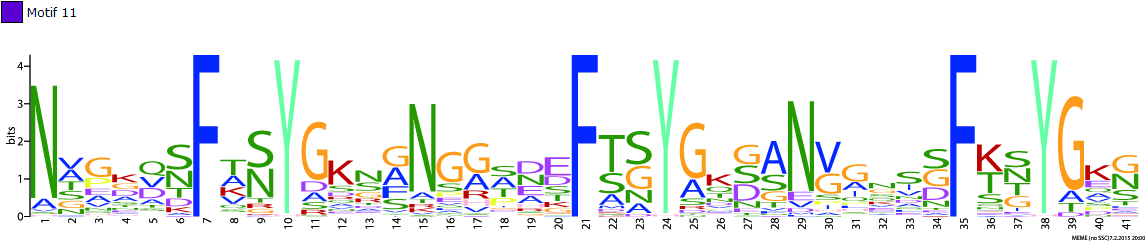


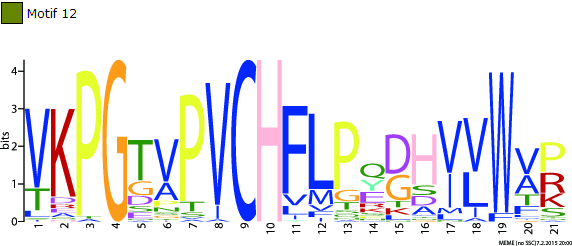


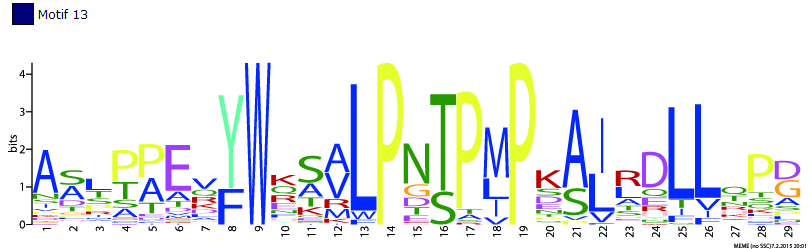


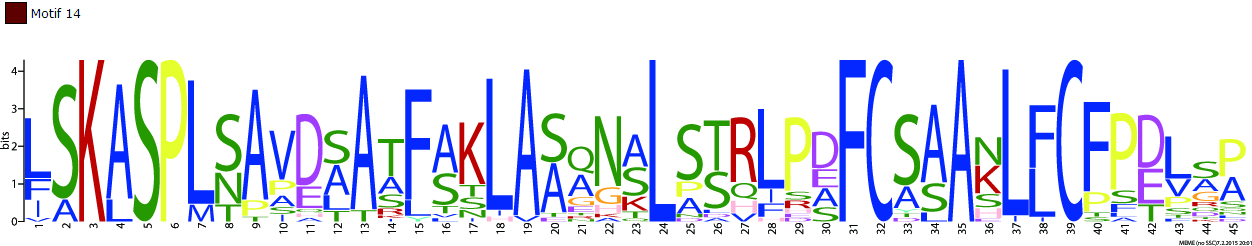

Supplement: Supplementary file 8 — Data S6. Schematic of motifs of BURP domain‐containing proteins. The schematic was derived from MEME. The order of motifs of the BURP domain‐containing proteins in the schematic was automatically generated by MEME according to scores. [file ECE3-5-5394-s008.doc]
